# Genome sequence of *Shigella* phage vB_EcoM_SS01

**DOI:** 10.1128/mra.00177-26

**Published:** 2026-06-04

**Authors:** Joseph M. O’Brien, Narima Eerqing, Serge Mostowy, Zoe A. Dyson

**Affiliations:** 1 Department of Infection Biology, Faculty of Infectious and Tropical Diseases, London School of Hygiene and Tropical Medicine, London, United Kingdom; 2 Wellcome Sanger Institute, Wellcome Genome Campus 47665, Hinxton, United Kingdom; Queens College Department of Biology, Queens, New York, USA

**Keywords:** bacteriophage, phage, whole genome sequencing, *Shigella*, *Shigella sonnei*, *Escherichia coli*, genome analysis, nanopore

## Abstract

We present the genome of phage vB_EcoM_SS01, an obligately lytic phage infective for *Shigella sonnei* isolated on strain 391324 from a wastewater sample collected in London, United Kingdom. The genome sequence is 88,160 bp with 149 annotated genes and a G + C content of 38.93%.

## ANNOUNCEMENT

Obligately lytic phages kill their host bacteria following replication, making them potentially valuable biocontrol agents for antimicrobial-resistant pathogens. *Shigella sonnei* are a major cause of diarrheal disease globally and recognized as World Health Organization priority pathogens due to fluoroquinolone resistance (1–3).

Phage vB_EcoM_SS01 was isolated at 37°C overnight using Tryptic Soy Broth (TSB; 10 mL), wastewater (2 mL; 0.22 μM filtered mixed liquor from Mogden municipal plant, London, United Kingdom, 2024), and exponential-phase bacterial culture (200 μL; *S. sonnei* strain 391324; genotype 1.5) (4, 5) via standard enrichment methods (6, 7). Using Tryptic Soy Agar supplemented with 0.01% Congo Red to select for virulent *S. sonnei* carrying the pINV invasion plasmid (8), a lawn plate inoculated via a single colony of 391324 was exposed to a 10 μL drop of filtered (0.22 μM) enrichment culture prior to incubation at 37°C overnight. Single plaques were excised and resuspended in 500 μL of TSB, followed by purification through five rounds of dilution and reisolation using 10 μL drops of a 10-fold dilution series (6, 7).

Following RNAse and DNAse treatment (5 μL each; 10 μg/mL), gDNA was extracted following Polyethylene Glycol 8000 precipitation (9, 10) of 5 mL of phage lysate using the GenFind V3 DNA Extraction Kit (Beckman Coulter), with modifications as follows. Phage pellets were resuspended in 400 μL lysis buffer containing 9 μL Proteinase K (96 mg/mL), and RNase A was omitted (11). Unsheared gDNA was prepared for sequencing with an R10.4.1 flow cell using V14 rapid barcoding and sequencing kits and sequenced via the MinION Mk1B platform (Oxford Nanopore Technologies) as per the manufacturer’s instructions. Adaptor trimming and read quality binning were performed using MinKNOW v6.0.14. Super Accuracy basecalling was performed on 210,567 raw reads with an *N*_50_ of 8,435 bp using Dorado v1.0.2 (12), prior to downsampling to 40% of the total reads with SeqKit v2.9.0 (13) for assembly with Autocycler v0.5.2 (14). Autocycler yielded a single consensus contig with >4,000-fold average read depth. Genome annotation was conducted with PHAROKKA v1.8.1 (15), followed by Phold v1.0.0 (16). Suitability for phage therapy was assessed with PhageLeads (17), lifecycle predicted with PhaTYP via PhaBOX v2.0 (18), and taxonomic classification performed with TaxMyPhage v3.3.6 (19). Genome structure was assessed by mapping raw reads to the assembled contig with Bowtie v2.2.4 (20) and visualization with IGV v2.17.4 (21). Unless otherwise stated, default parameters were used.

**Fig 1:**
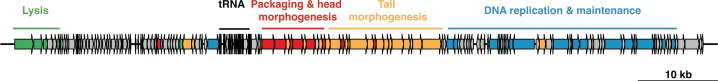
Linear genome map of *Shigella* phage vB_EcoM_SS01. Arrows indicate coding sequences which are colored by putative phage functions (as labeled). Genes with other or unknown functions are colored gray.

Phage vB_EcoM_SS01 has a linear, circularly permuted dsDNA genome of 88,160 bp and a G + C content of 38.93%. It has 149 putative genes, with 70% encoded in the forward orientation and 43% assigned a putative function (Fig. 1). A cluster of 24 tRNAs was identified that might represent an adaptation to codon bias in the *Shigella* host, which typically has a G + C content of ~50% (4). Phage vB_EcoM_SS01 is a *Felixounavirus*, most closely related to *Escherichia* phage vB_EcoM_LMP25 (93.89% average nucleotide identity; accession number MK482688). DNA metabolism genes included a DNA polymerase (CDS_116), DNA helicase (CDS_121), and exonuclease (CDS_125). Genes associated with virion morphogenesis included a portal (CDS_068), major head (CDS_073), tail sheath (CDS_078), tail assembly chaperones (CDS_066, CDS_080-081), baseplate subunits (CDS_085-089), and tail fiber proteins (CDS_069, CDS_091-092). Identifiable lysis genes included multiple endolysins (CDS_003, CDS_050) and an Rz-like spanin (CDS_006). As with other *Felixounaviruses*, RIIA and RIIB genes were detected (CDS_001-002), but their potential role in lysis inhibition remains unclear (22–25). No genes associated with lysogeny, antimicrobial resistance, or virulence were observed, suggesting potential utility of phage vB_EcoM_SS01 in controlling *S. sonnei*.

## Data Availability

The assembled sequence of phage vB_EcoM_SS01 has been deposited in GenBank under accession number PX682358. Raw reads have been deposited in the Sequence Read Archive under accession number SRR37336928.
